# Moderate-intensity exercise training uniquely modulates circulating lipid species beyond classical lipid levels in humans

**DOI:** 10.1016/j.ebiom.2025.105849

**Published:** 2025-07-15

**Authors:** Yu Zhang, Zhengzheng Zhang, Borja Martinez-Tellez, Xinyu Di, Alida Kindt, Isabelle Kohler, Francisco J. Osuna-Prieto, Charles Clark, Nicolas Drouin, Amy Harms, Thomas Hankemeier, Jonatan R. Ruiz, Lucas Jurado-Fasoli

**Affiliations:** a Metabolomics and Analytics Centre, Leiden Academic Centre for Drug Research (LACDR), Leiden University, the Netherlands; b Department of Physical Education and Sports, Faculty of Sport Sciences, Sport and Health University Research Institute (iMUDS), University of Granada, Carretera de Alfacar s/n, Granada, 18071, Spain; c CIBER de Fisiopatología de la Obesidad y Nutrición (CIBEROBN), Instituto de Salud Carlos III, Madrid, Spain; d Department of Nursing, Physiotherapy and Medicine, SPORT Research Group (CTS-1024), CIBIS Research Center, University of Almería, Spain; e Division of BioAnalytical Chemistry, Department of Chemistry and Pharmaceutical Sciences, Amsterdam Institute of Molecular and Life Sciences (AIMMS), Vrije Universiteit Amsterdam, Amsterdam, the Netherlands; f Hospital Universitari Joan XXIII de Tarragona, Institut d'Investigació Sanitària Pere Virgili (IISPV), Tarragona, Spain; g CIBER de Diabetes y Enfermedades Metabólicas Asociadas (CIBERDEM)-Instituto de Salud Carlos III (ISCIII), Madrid, 28029, Spain; h Instituto de Investigación Biosanitaria, Ibs.Granada, Granada, Spain; i Department of Physiology, Faculty of Medicine, Sport and Health University Research Institute (iMUDS), University of Granada, Granada, Andalucía, Spain

**Keywords:** Concurrent training, Lipidomics, Metabolism, Precision medicine, Metabolic health

## Abstract

**Background:**

Regular physical exercise shows significant health-related benefits, potentially through the modulation of lipid metabolism in an intensity-dependent manner.

**Methods:**

In this study, we profiled 794 plasma lipid species across 18 subclasses following a 24-week supervised concurrent and randomised exercise intervention at moderate and vigorous intensities in 101 young, sedentary adults.

**Findings:**

Here, we demonstrate that moderate-intensity exercise, but not vigorous-intensity, significantly increased plasma levels of glycerophospholipids and triacylglycerol species. Interestingly, we also identified a sex-specific response to moderate-intensity exercise, with men exhibiting elevated glycerophospholipids and lysophospholipids species, and women showing significant increases in triacylglycerols species. Increments in glycerophospholipids species were associated with improvements in cardiorespiratory fitness, i.e., VO_2_peak.

**Interpretation:**

Importantly, while traditional lipid markers, including total cholesterol or triglycerides remained unchanged after the exercise intervention, our findings suggest that exercise partially exerts its health benefits by selectively targeting and modifying specific lipid subtypes in an intensity, sex-dependent manner.

**Funding:**

The study was supported by the 10.13039/501100011011Junta de Andalucía, 10.13039/100020230Consejería de Transformación Económica, Industria, Conocimiento y Universidades Dirección General de Investigación y Transferencia del Conocimiento (ref. P18-RT-4455, ref. SOMM17/6107/UGR, and DOC 01151) and European Regional Development Funds (ERDF), the 10.13039/501100003329Spanish Ministry of Economy and Competitiveness via the Fondo de Investigación Sanitaria del Instituto de Salud Carlos III (PI13/01393), and PTA-12264, Retos de la Sociedad (DEP2016-79512-R), the 10.13039/100019805Fundación Iberoamericana de Nutrición (FINUT), the Redes Temáticas de Investigación Cooperativa RETIC (Red SAMID RD16/0022), the AstraZeneca HealthCare Foundation, the University of Granada Plan Propio de Investigación 2016 Excellence actions: Unit of Excellence on Exercise and Health (UCEES).

**Research in context**

**Evidence before this study**

Regular physical exercise improves metabolic health, cardiorespiratory fitness, and reduces the risk of chronic diseases. These benefits are partially mediated by changes in lipid metabolism. However, most studies to date have focused on classical lipid markers such as total cholesterol and triglycerides, with limited exploration of how exercise affects a broader spectrum of lipid species.

**Added value of this study**

This study provides a comprehensive lipidomic analysis of the effects of a 24-week supervised concurrent exercise intervention (moderate- vs. vigorous-intensity) in young, sedentary adults. Using targeted mass spectrometry, we quantified nearly 800 plasma lipid species. We demonstrate that moderate-intensity exercise significantly increases levels of glycerophospholipids, lysophospholipids, and triacylglycerol species, whereas vigorous-intensity exercise induces fewer changes. Importantly, these changes were linked to improvements in cardiorespiratory fitness, especially among fitness responders. The study also revealed sex-specific responses in triacylglycerol levels and provided insights into the molecular lipid pathways modulated by exercise.

**Implications of all the available evidence**

These findings highlight that moderate-intensity exercise training induces specific lipidomic adaptations beyond classical lipid markers, particularly in glycerophospholipids and triacylglycerols, which may contribute to exercise-induced health benefits. Our results support the use of advanced lipidomic profiling to better understand the molecular mechanisms underlying the benefits of exercise. This approach can help identify new lipid biomarkers for monitoring training responses and developing personalised exercise interventions based on individual lipidomic signatures.

## Introduction

Regular physical exercise is related to a reduced risk of all-cause mortality^1^ and a lower prevalence of chronic diseases,^2^^,^^3^ largely due to its beneficial effects on cardiometabolic health, physical function, and cardiorespiratory fitness.^4^^,^^5^ However, the molecular mechanisms underlying its health-related benefits are not fully understood.

Exercise health-related benefits are partly explained by its effects on lipid metabolism across multiple tissues.^6^ Exercise is a non-pharmacological intervention that can oxidise fats and carbohydrates simultaneously as fuel sources depending on the duration and intensity.^7^ Acute moderate-intensity exercise increases the release of free fatty acids from adipose tissue to contribute to increased energy expenditure during exercise, whereas chronic exercise augments lipid oxidation in the skeletal muscle and decreases the visceral storage of adipose tissue.^6^ Additionally, regular exercise improves cholesterol and triacylglycerol (TG) circulatory levels, contributing to better overall health and reductions in the risk of chronic diseases.^8^^,^^9^ Up to date, most studies investigating the effects of regular exercise on lipids have primarily focused on total lipoproteins and total cholesterol blood levels,^10^, ^11^, ^12^ with limited exploration of its impact on the broader spectrum of lipid classes and subclasses.

Lipids are hydrophobic or amphipathic small molecules that are typically classified into different categories, (e.g., glycerolipids, glycerophospholipids, sphingolipids, and sterols) and subclasses based on their head groups and the diversity of their fatty acid chains.^13^^,^^14^ They play critical roles in maintaining cellular homoeostasis, acting as signalling molecules and second messengers, with functions that may vary depending on the specific lipid species.^15^, ^16^, ^17^ Notably, our recent findings demonstrated that our long-term moderate-intensity exercise intervention selectively reduced the levels of oxidised lipids derived from omega-6 oxidised lipids, without affecting omega-3 species.^18^ These findings suggest that changes in overall lipid levels may not fully capture the health benefits of exercise, as each lipid subtype has unique biological functions.

For instance, we showed that while total TG levels remained unchanged following acute cold exposure, dynamic changes were observed across TG subtypes.^19^ Similarly, we found that a 24-week supervised exercise program in young adults reduced fat mass and improved cardiorespiratory fitness without significantly altering total cholesterol or total TG levels.^20^ Given the complexity and diverse functions of different lipid species, understanding their specific changes in response to exercise is crucial for uncovering the molecular mechanisms that underpin the health-related benefits of exercise training in humans.

This study aimed to investigate the effects of a 24-week supervised concurrent exercise intervention (i.e., combining endurance and resistance) at moderate and vigorous intensities on plasma lipid species in young, sedentary adults. Using advanced mass spectrometry (MS) based lipidomic techniques, we identified and quantified a comprehensive profile of approximately 800 distinct lipid species. Our findings underscore the importance of moderate-intensity exercise in modulating lipid species beyond traditional lipid measures, providing deeper insights into the molecular mechanisms underlying the health benefits of exercise.

## Methods

### Study participants

**Fig. 1:**
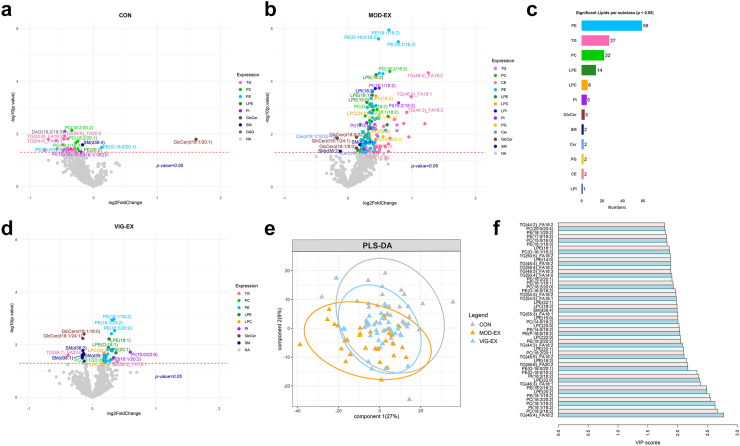
**Effects of 24 weeks of supervised exercise training on plasma lipid species levels.** (a, b, d): Volcano plots illustrating the changes in lipid species in the control group (a, n = 35), moderate-intensity exercise group (b, n = 32), and vigorous-intensity exercise group (d, n = 34). The *x*-axis represents the log2 fold change, whereas the *y*-axis represents the statistical significance (−log10 P value). P values were obtained from a paired t-test. c: Number of significant lipid species per lipid class that increased after intervention in the moderate-intensity exercise group. e: Partial least square discriminant analysis (PLS-DA) classifying the three exercise groups based on the plasma levels of lipid species. f: Variable importance in projection (VIP) plot highlighting the top 50 most important lipids species identified by PLS-DA. *Abbreviations*: CE, cholesterol ester; CON, control group; DAG, diacylglycerol; HexCer, hexosylceramide; LPC, lysophosphatidylcholine; LPE, lysophosphatidylethanolamine; LPI, lysophosphatidylinositol; MOD-EX, moderate-intensity exercise group; PC, phosphatidylcholines; PC-O, alkyl substituent phosphatidylcholines; PE, phosphatidylethanolamine; PE-O, alkyl substituent phosphatidylethanolamine; PE-P, alkenyl substituent phosphatidylethanolamines; PG, phosphatidylglycerol; PI, phosphatidylinositol; PLS-DA, partial least square discriminant analysis; PS, phosphatidylserine; TG, triacylglycerol; VIG-EX, vigorous-intensity exercise group.

This study was conducted under the framework of the ACTIBATE (ACTivating Brown Adipose Tissue through Exercise; ClinicalTrials.gov ID: NCT02365129; Fig. 1) randomised controlled trial.^21^ The ACTIBATE study included 145 sedentary Caucasian males and females, aged between 18 and 25 years, which were recruited from Granada (Spain) through social networks, local media, and posters. Inclusion criteria were reporting to be sedentary (i.e., <20 min/day of moderate-to-vigorous physical activity for <3 days/week), not taking any medication, being a non-smoker, and having a stable body weight for the past 3 months. Exclusion criteria included having been diagnosed with diabetes, hypertension, or other significant medical conditions that could interfere with or be aggravated with exercise, being pregnant, using medications affecting energy metabolism, or being frequently exposed to cold temperatures. While the study population may not perfectly reflect the full diversity of young adults worldwide, the inclusion criteria (age, sedentary status, absence of chronic disease) and the recruitment strategy allow us to consider the sample reasonably representative of healthy young sedentary adults within an urban European context. Participants self-reported their sex (male or female) during the enrolment process. Sex was not used as an inclusion or exclusion criterion. However, both males and females were included in the study and distributed across intervention groups. Sex-stratified analyses were conducted for key outcome variables.

### Study design

The current study includes secondary analyses from the single-centre ACTIBATE randomised controlled trial, of which the detailed design is described elsewhere.^21^ All participants provided written informed consent. The study was conducted at the Sport and Health University Research Institute and the Virgen de las Nieves University Hospital of the University of Granada. It was carried out over two consecutive years in four waves (i.e., from September 2015 to June 2016, and from September 2016 to June 2017), and concluded at the end of the exercise intervention period.

After baseline examination, participants were randomly assigned to three groups via computer-generated simple unrestricted randomisation^22^: (i) a control group (no exercise, CON), (ii) a moderate-intensity exercise group (MOD-EX), and (iii) a vigorous-intensity exercise group (VIG-EX). Assessment staff and data analysts will be blinded to participant randomisation assignment. Participants were informed of their group assignments with no delay between randomisation and the start of the intervention. To maintain the trial's internal and external validity, stringent standardisation procedures were applied to both data collection and the intervention process. All outcome assessments were carried out at the same time of day both before and after the 24-week intervention. Participants were advised to maintain their usual routines, including physical activity and dietary habits over the study period. No important changes were performed in the methodology or outcomes after the beginning of trial and no relevant adverse events were recorded.

### Supervised exercise training program

A comprehensive description of the supervised exercise training program used in this study, which combined resistance and endurance training as recommended by World Health Organization (WHO) guidelines,^23^ has been published elsewhere.^21^ Participants trained at the same training centre 3–4 times per week for 24 weeks, completing a total of 150 min/week of endurance exercise (performed in all sessions) and 80 min/week of resistance (performed during 2 sessions/week) exercise. The MOD-EX group conducted the endurance training at 60% of heart rate reserve (HRres) and performed the resistance training at 50% of the repetition maximum (RM). The VIG-EX group performed the endurance training at 60% of HRres for 75 min/week, and 80% of HRres for 75 min/week and conducted the resistance training at 70% of their RM.

The load for resistance exercises was individually and monthly adjusted. For the MOD-EX group, participants began with a four-week familiarisation phase, during which exercise intensity was progressively increased up to approximately 50%–60% of their 1-RM. After this initial phase, participants continued training at a moderate intensity (50%–60% of 1-RM) throughout the intervention. To ensure individualised progression, 1-RM tests were performed monthly for key resistance exercises (such as the bench press and leg press), and training loads were adjusted accordingly. This approach allowed participants to maintain the intended relative intensity as their strength improved, while ensuring safety and effective adaptation. For the VIG-EX group, a similar familiarisation phase was conducted. However, following familiarisation, exercise intensity was progressively increased from 60% to 80% of 1-RM. The gradual increase was achieved by incrementally adjusting the training load each week, targeting 5%–10% increases based on participants' perceived exertion, performance, and technical proficiency. As in the MOD-EX group, 1-RM assessments were conducted monthly to individually update the training loads, ensuring participants consistently worked within the desired vigorous intensity range.

Participants exercised in groups of 10–12 simultaneously each day throughout the 24-week intervention. Attendance and adherence to the prescribed endurance training intensity were registered using a heart rate monitor (RS800CX, Polar Electro Öy, Kempele, Finland).

Participants in the MOD-EX and VIG-EX included in the current study attended on average 85.72% and 86.22% of prescribed sessions respectively.

### Blood sample collection

Blood samples were collected in the morning after a 10-h overnight fast; 1–3 weeks before the exercise intervention, and 3–4 days after its completion. The samples were collected using EDTA-coated Vacutainer® Hemogard™ tubes and immediately centrifuged to obtain plasma. The plasma samples were then aliquoted and stored at −80 °C until analysis.

### Determination of plasma levels of lipid species

Plasma and quality control (QC) samples were randomised into three batches separately for sample preparation and lipidomics data acquisition. Before sample preparation, an internal standard (IS) mix was added to each sample, containing SPLASH® LIPIDOMIX® Mass Spec Standard, lysophosphatidylserines (LPS) 17:1, lysophosphatidylinositols (LPI) 17:1, lysophosphatidylglycerols (LPG)17:1 (Avanti polar lipids), deuterated lactosylceramides (LacCer) d18:1/16:0-d_9_-ISTD, Ceramide (Cer) LIPIDOMIX® 330713 Avanti, and hexosylceramides (HexCer) d18:1/16:0-d_9_-ISTD. Lipids were extracted using liquid-liquid extraction with an extraction solvent mixture composed of methyl tert-butyl ether, methanol, and water (10:3:2.5, *v/v/v*). The mixture of extraction solvents and plasma was then incubated at room temperature for 10 min and centrifuged at 15,800 rcf for 10 min. The supernatant (520 μL) was collected, evaporated in a vacuum concentrator to dryness, and reconstituted in 100 μL of a mixture of acetonitrile: methanol (3:7, *v/v*). The reconstituted mixture was vortexed and centrifuged for 10 min. Finally, the supernatant was injected into the liquid chromatography-mass spectrometer (LC-MS) for analysis.

Lipid species were profiled using a validated hydrophilic interaction liquid chromatography-tandem mass spectrometry (HILIC-MS/MS) platform. The validated method enabled the determination of approximately 1200 lipid species across 18 subclasses.^24^ Targeted lipid profiling was performed on a QTRAP 6500+ mass spectrometer (SCIEX, Concord, ON, Canada) coupled to an Exion LC AD system (SCIEX, Concord, ON, Canada). The separation was performed using a Phenomenex Luna® amino column (100 mm × 2 mm, 3 μm, Phenomenex). Ionisation was achieved using a Turbo V source equipped with an electrospray ionisation probe. Three different acquisitions, using both negative and positive modes, were performed on one sample to identify lipids and ensure adequate sensitivity (Table S1).

The acquired MS data was evaluated using SCIEX OS Software (version 2.1.6) for quantitative analysis. Assigned multiple reaction monitoring peaks were integrated and the data was normalised using one IS per subclass. To assess data quality, we examined the relative standard deviation (RSD) of target analytes in QC samples, which consisted of pooled study samples, as well as the background signal. Target analytes with RSDs exceeding 30% in QC samples or 40% for the background signal were excluded from statistical analysis.^25^ Thirty-six QC samples from the three batches clustered distinctly in the three acquisition methods (Figure S2a–c). After removing outliers, 794 lipids across 18 subclasses were quantified (Figure S2e). These subclasses included cholesterol esters (CE), Cer, diacylglycerols (DG), glucosylceramides (GlcCer), HexCer, LacCer, lysophosphatidylcholines (LPC), lysophosphatidylethanolamines (LPE), LPG, LPI, LPS, phosphatidylcholines (PC), phosphatidylethanolamines (PE), phosphatidylglycerols (PG), phosphatidylinositols (PI), phosphatidylserines (PS), sphingomyelins (SM), and TG.

### Anthropometric and body composition measurements

Participants' weight and height were measured without shoes and wearing light clothing using a 799 SECA electronic column scale and stadiometer (Hamburg, Germany). From these measurements, the body mass index (BMI; kg/m^2^) was calculated. Lean body mass, fat mass, and visceral adipose tissue (VAT) mass were assessed using dual-energy X-ray absorptiometry (DXA) with a Discovery Wi device (Hologic Inc., Bedford, MA, USA) and analysed with APEX software (version 4.0.2). Body fat percentage was reported as the proportion of total body weight attributed to fat mass.

### Physical fitness

All participants underwent physical fitness assessments during two sessions following a previously described methodology.^20^

The muscle strength of the subjects was assessed using grip strength, leg press, and bench press tests. Grip strength was measured with a Takei 5401 digital Grip-D handheld dynamometer (Takei, Tokyo, Japan), where participants gradually and continuously squeezed the grips until maximum strength was reached. Following a systematic warm-up, the 1-RM leg press and bench press tests were conducted using a leg press machine (A300 Leg Press, Model 2531, Keiser Corporation, Fresno CA, USA) and a bench within a pneumatic power rack (Power rack, Model 3111, Keiser Corporation, Fresno CA, USA), respectively. The 1-RM of both exercises was estimated by the equation previously proposed.^26^

To assess the subjects' maximal oxygen consumption, a modified Balke protocol was employed on a treadmill.^27^ Respiratory gas exchange, including oxygen consumption (VO_2_) and carbon dioxide production (VCO_2_), was measured using indirect calorimetry throughout the test with a CPX Ultima CardioO2 metabolic cart (Medical Graphics Corp, St. Paul, MN, USA). VO_2_peak was determined as the highest observed VO_2_ value, after excluding significant artifacts if needed. VO_2_peak was expressed relative to body weight. Time to exhaustion was measured in seconds.

### Cardiometabolic risk factors

Biochemical evaluations included glucose, total cholesterol (TC), high-density lipoprotein cholesterol (HDL-C), and TG, all measured using a Beckman Coulter AU5832 autoanalyzer (Brea, CA, USA). Low-density lipoprotein cholesterol (LDL-C) levels were calculated using the Friedewald formula. Insulin concentrations were determined with the Access Ultrasensitive Insulin Chemiluminescent Immunoassay Kit (Beckman Coulter). The homoeostasis model assessment (HOMA) index was calculated as insulin (μU/mL) × glucose (mmol/L)/22.5.

Additionally, liver enzymes and cardiometabolic risk factors, including glutamate pyruvate transaminase (GTP), gamma-glutamyl transferase (GGT), alkaline phosphatase (ALP), apolipoproteins A and B were measured using an AU5832 spectrophotometer (Beckman Coulter) with Beckman Coulter reagents (OSR6507, OSR6520, OSR6204, 446410, and 447730).

Leptin and adiponectin levels were measured in plasma using the MILLIPLEX MAG Human Adipokine Magnetic Bead Panel 2 (Cat. No. HADK2MAG-61K) and the MILLIPLEX MAP Human Adipokine Magnetic Bead Panel 1 (Cat. No. HADK1MAG-61K), respectively, from Luminex Corporation (Austin, TX, USA).

Resting blood pressure was recorded in a sitting position twice over three consecutive days using an Omron M2 automatic sphygmomanometer (Omron Healthcare, Kyoto, Japan). Three measurements were taken each time and the average of the three values was used for further analysis.

### Ethics

The study protocol and experimental design were applied following the last revised ethical guidelines of the Declaration of Helsinki. The study was approved by the Ethics Committee on Human Research of the University of Granada (no. 924) and the Servicio Andaluz de Salud (Centro de Granada, CEI-Granada); all participants gave informed consent.

### Statistical analysis

This study includes secondary analyses from a randomised controlled trial aimed at determining the effects of a 24-week supervised exercise intervention on brown adipose tissue, which was originally powered to detect changes in the primary outcome. Therefore, no specific power calculation was performed. Participants with blood sample determinations were included in this secondary analysis. However, we conducted post hoc power analyses to assess the robustness of our findings across key comparisons. Using five representative lipid species in each analysis based on variable importance in projection (VIP), we estimated effect sizes (Cohen's f for ANOVA and d for t-tests) and calculated statistical power at a significance level of α = 0.05 using the pwr package (v.1.3.0).

All statistical analyses were conducted using R software (version 4.2.2). Descriptive data are expressed as mean ± standard deviation unless otherwise stated. Before data analysis, we pre-processed the lipidomics data to ensure suitability for analysis. Initially, the data distribution was assessed using Shapiro–Wilk tests and Q-Q plots. Consequently, the data were log2-transformed. Missing-not-at-random (MNAR) values were evaluated using Fisher's exact tests when the proportion of missing data exceeded 20% for any lipid species Finally, conserved missing values were then imputed using quantile regression imputation of left-censored data (QRILC)^28^ with the R package imputeLCMD (v.2.1). To assess the robustness of the imputation approach, sensitivity analyses were performed using alternative methods, including k-nearest neighbours (KNN) and half-minimum imputation, via the VIM package (v.6.2.2). In our study, loss to follow up and missing lipidomic data occurred primarily due to participants not meeting the ≥70% adherence threshold or due to technical limitations in lipidomic sample quality. These factors are unrelated to the lipidomic outcomes themselves and were not systematically associated with baseline lipid profiles, group allocation, or other key variables. Therefore, we consider the missing data to be Missing At Random (MAR), as the reasons for missingness are explainable by observed variables (e.g., adherence, sample quality) and not by unobserved outcomes.

All the data analysed are presented as the log2 fold change (log2FC) relative to the baseline. Paired t-tests were performed to determine lipid species changes within groups after exercise intervention using the matrixTests (v.0.2.3) package. Partial least square discriminant analysis (PLS-DA) and VIP analysis were used to analyse the differences among groups and the key lipid species that affected the differences between the groups. PLS-DA and VIP analysis were conducted using the mixOmics package (v.6.22.0). Additionally, Principal Component Analysis (PCA) was performed with the factoextra package (v.1.0.7) to characterise lipidomic shifts in the vigorous-intensity exercise group. Between-group differences in lipid species were assessed using a one-way analysis of variance (ANOVA) followed by Tukey's HSD post hoc comparison and Bonferroni corrections. These analyses were also conducted on men and women separately. Baseline sex differences in lipid species were evaluated using independent two-sample t-tests with the stats package (v.4.5.0). Analysis of covariance (ANCOVA) adjusting for baseline data was used to compare changes in classical lipid makers (i.e., TG and TC) across the three groups after exercise intervention. Both ANOVA and ANCOVA analyses with post-hoc comparisons were performed with the rstatix (v.0.7.2), emmeans (v.1.10.6), and multcomp (v.1.4.26) packages.

Lipid Ontology (LION)-term enrichment analysis was employed to identify lipid pathways significantly enriched after the long-term exercise training.^29^ In this analysis, a one-way ANOVA F-test was used to compare the three groups, while one-tailed Welch's two-sample t-tests were applied for comparisons between two conditions (i.e., MOD-EX vs. CON, VIG vs. CON, MOD-EX vs. VIG-EX, and VIG-EX vs. MOD-EX). The distributions of associated LION-terms across the ranked list were evaluated against uniform distributions using one-tailed Kolmogorov–Smirnov tests. The Lipid Ontology–LION/web and R package *shiny* (v.1.10.0) were used for the enrichment analysis.

A within-individual change distribution was calculated to determine participants who experienced a clinically meaningful change from baseline to post-intervention in VO_2_peak relative to body weight and fat mass percentage. Participants were grouped as responders if the standardised effect size of the intervention was equal to or exceeded a Cohen of 0.20 or as non-responders if this value was less than 0.20.^30^ We compared changes in lipid species after the intervention (log2FC) between responders and non-responders in VO_2_peak relative to body weight and fat mass percentage, as well as their overlap, using ANCOVA, including the intervention group as covariates. In addition, we calculated a z-score for each participant of all lipid species that significantly increased after the intervention in the MOD-EX. A Pearson correlation analysis was performed between the mean z-scores and changes in physical fitness, followed by a partial correlation analysis adjusted for sex using the *corrplot* (v.0.95) and *ppcor* (v.1.1) packages. Results were considered statistically significant at values P < 0.05.

### Role of funders

Funders did not participate in the study design, data collection, data analyses, interpretation, or writing of the manuscript.

## Results

Out of the initial 145 participants assigned to one of the three groups for the exercise training, 101 participants were included in the analysis (Figure S1). A total of 44 participants were excluded from the main analysis due to not completing the study (i.e., attending <70% of the total training sessions; n = 31) or lacking valid lipidomic measurements (n = 13). While this dropout rate is substantial, attrition was balanced across the three intervention groups and showed no apparent pattern in relation to sex, age, or baseline VO_2_peak. No adverse events or unintended effects were reported in either group during the intervention period.

The phenotypical traits of the participants included in these secondary analyses are listed in Table 1. After the 24-week exercise intervention, both MOD-EX and VIG-EX decreased adiposity (i.e., fat mass percentage Δ = −2.9 ± 2.5% and Δ = −3.3 ± 3.4% respectively; visceral adipose tissue Δ = −45 ± 25 g and Δ = −67 ± 91 g respectively) and increased cardiorespiratory fitness (i.e., VO_2_peak relative to body weight, Δ = 4.5 ± 6.2 mL/kg/min and Δ = 4.7 ± 5.0 mL/kg/min respectively; VO_2_peak in absolute terms, Δ = 304 ± 369 mL/min and Δ = 285 ± 375 mL/min respectively) compared to the CON group. However, no significant changes were observed in classical lipid markers (i.e., TC, HDL-C, LDL-C, total TG, APOA1, and APOB) across the three groups (CON, MOD-EX, and VIG-EX; all P > 0.05; Table S2).

**Table 1:** Baseline characteristics of the study participants.

Variables	N	All	N	CON	N	MOD-EX	N	VIG-EX
		Mean (SD)		Mean (SD)		Mean (SD)		Mean (SD)
Age (years)	101	22.2 (2.2)	35	22.3 (2.0)	32	22.2 (2.1)	34	22.2 (2.5)
Sex (n, % female)	101	69 (68.3%)	35	21 (60.0%)	32	24 (75.0%)	34	24 (70.6%)
**Anthropometric and body composition**								
BMI (kg/m^2^)	101	24.7 (4.2)	35	24.0 (3.7)	32	25.2 (4.3)	34	25.2 (4.4)
Lean body mass (kg)	101	41.4 (9.2)	35	41.5 (10.2)	32	40.6 (7.7)	34	42.0 (9.5)
Lean mass index (kg/m^2^)	101	14.5 (2.2)	35	14.5 (2.4)	32	14.4 (1.9)	34	14.7 (2.4)
Fat mass (%)	101	35.9 (7.4)	35	34.1 (7.5)	32	37.3 (7.8)	34	36.5 (6.7)
Fat mass index (kg/m^2^)	101	8.8 (2.8)	35	8.1 (2.5)	32	9.4 (3.2)	34	9.1 (2.6)
Visceral adipose tissue (g)	101	346.3 (172.8)	35	316.3 (148.6)	32	364.1 (184.6)	34	360.3 (185.1)
Waist circumference (cm)	99	81.1 (12.6)	35	79.8 (12.5)	31	81.6 (11.9)	33	82.1 (13.6)
**Physical fitness**								
Handgrip strength (kg)	88	62.2 (15.5)	28	62.2 (15.5)	30	60.6 (15.2)	30	62.0 (15.3)
Leg press (kg)	88	197.5 (62.7)	28	202.3 (65.7)	30	190.1 (55.9)	30	200.5 (67.4)
Bench press (kg)	88	30.8 (13.7)	28	33.5 (17.0)	30	28.3 (10.5)	30	30.8 (13.0)
Time to exhaustion (s)	89	926.2 (206.6)	31	897.4 (242.5)	31	935.0 (193.2)	27	949.3 (178.5)
VO_2_peak (mL/kg/min)	99	41.1 (8.4)	33	42.7 (9.6)	32	40.4 (6.7)	33	40.2 (8.7)
**Cardiometabolic risk parameters**								
Systolic blood pressure (mmHg)	100	117.0 (11.2)	34	116.5 (12.0)	32	117.9 (10.7)	34	116.8 (11.3)
Diastolic blood pressure (mmHg)	100	71.1 (7.3)	34	69.3 (7.6)	32	68.9 (62.9)	34	72.0 (8.1)
Glucose (mg/dL)	101	87.2 (6.3)	35	86.9 (5.9)	32	88.1 (6.9)	34	86.8 (6.2)
Insulin (μIU/mL)	101	8.1 (4.0)	35	7.6 (3.4)	32	8.6 (4.6)	34	8.3 (4.1)
HOMA-IR	101	1.8 (1.0)	35	1.6 (0.8)	32	1.9 (1.2)	34	1.8 (1.0)
GTP (IU/L)	101	18.6 (18.0)	35	21.3 (27.9)	32	15.7 (8.4)	34	18.5 (9.6)
GGT (IU/L)	101	19.1 (20.2)	35	24.2 (32.4)	32	15.5 (6.1)	34	17.3 (8.6)
ALP (IU/L)	101	72.4 (19.3)	35	71.3 (22.5)	32	72.0 (18.6)	34	74.1 (16.6)
TC (mg/dL)	101	162.9 (31.2)	35	153.8 (29.6)	32	164.4 (30.5)	34	170.9 (31.7)
HDL-C (mg/dL)	101	52.5 (11.6)	35	53.3 (9.5)	32	52.2 (11.9)	34	51.9 (13.3)
LDL-C (mg/dL)	101	94.3 (26.2)	35	85.8 (25.1)	32	96.7 (28.0)	34	100.9 (23.7)
TG (mg/dL)	101	84.2 (49.3)	35	73.8 (34.2)	32	88.4 (63.0)	34	90.9 (47.4)
APOA1 (mg/dL)	90	145.7 (29.4)	32	148.3 (19.6)	27	145.7 (36.2)	32	143.0 (31.8)
APOB (mg/dL)	90	68.6 (21.2)	32	63.0 (18.8)	27	68.4 (20.4)	32	74.4 (23.4)
Adiponectin (mg/L)	100	11.4 (7.6)	35	11.8 (6.9)	32	11.1 (8.3)	33	11.2 (7.7)
Leptin (μg/L)	100	6.3 (4.4)	35	5.5 (4.5)	32	7.0 (4.6)	33	6.4 (4.0)

*Abbreviations*: ALP, alkaline phosphatase; APOA1, apolipoprotein A-I; APOB, apolipoprotein B; BMI, body mass index; BAT, brown adipose tissue; CON, control group; GTP, guanosine triphosphate; GGT, gamma-glutamyl transferase; HDL-C, high-density lipoprotein cholesterol; HOMA-IR, homoeostasis model assessment of insulin resistance index; LDL-C, low-density lipoprotein cholesterol; MOD-EX, moderate-intensity exercise group; TC, total cholesterol; TG, triglyceride; VIG-EX, vigorous-intensity exercise group.

### Moderate-intensity exercise training increases glycerophospholipids and lysophospholipids species levels compared to the control group

First, we examined within-group lipid changes following the exercise intervention (Fig. 1). The MOD-EX group showed a significant increase in 145 lipid species plasma levels across twelve subclasses, with the highest number of lipid species and higher fold changes in plasma concentration observed for PE, TG, and PC (Fig. 1b and c). In contrast, both the CON group (n = 28 lipid species, Fig. 1a) and the VIG-EX group (n = 36 lipid species, Fig. 1d) exhibited minimal changes in lipid profiles. To investigate the variables that could explain the variability observed in component 1 of the PLS-DA model, participants were stratified by sex, cardiorespiratory fitness, and adiposity (Figure S3a, d, and g). We observed a clear separation along component 1 when stratifying by cardiorespiratory fitness and fatness but not by sex (Figure S3). Of note, we observed a high inter-individual variability in the lipidomic response in the VIG-EX group (Figure S4).

**Fig. 2:**
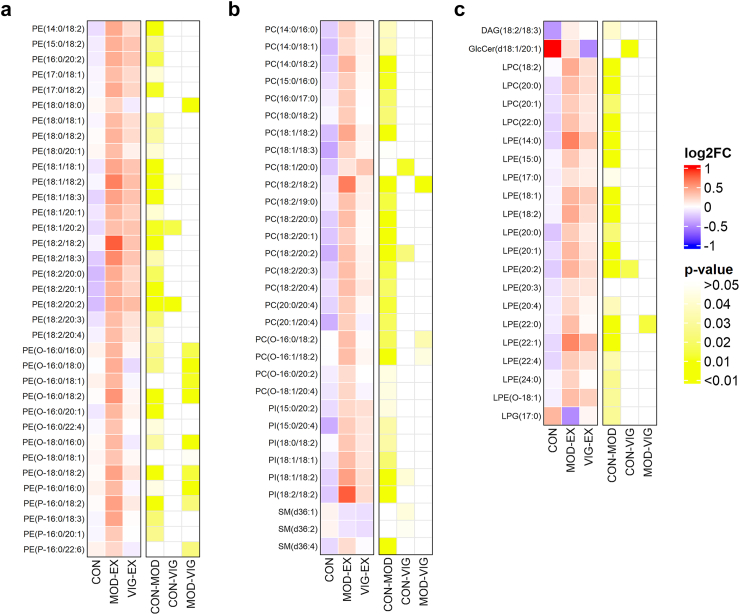
**24 weeks of moderate-intensity exercise training increases glycerophospholipid and lysophospholipid species levels compared to the control group.** The heatmap displays lipids (a: phosphatidylcholine; b: phosphatidylcholine, phosphatidylinositol and sphingomyelin; c: diacylglycerol, lysophosphatidylcholine, lysophosphatidylethanolamine, and lysophosphatidylglycerol) that exhibited significant changes after exercise intervention across the control, moderate-intensity, and vigorous-intensity exercise groups. The colour of each square (left columns) represents the log2 fold change relative to baseline for each lipid, and the colour of the yellow square (right columns) represents the P-value of the comparison between the groups. P-values were obtained using one-way ANOVA followed by Bonferroni correction. *Abbreviations*: CON, control group; DAG, diacylglycerol; GlcCer, glucosylceramide; LPC, lysophosphatidylcholine; LPE, lysophosphatidylethanolamine; LPE-O, alkyl substituent lysophosphatidylethanolamine; LPG, lysophosphatidylglycerol; MOD-EX, moderate-intensity exercise group; PC, phosphatidylcholine; PC-O, alkyl substituent phosphatidylcholines; PE, phosphatidylethanolamine; PE-O, alkyl substituent phosphatidylethanolamine; PE-P, alkenyl substituent phosphatidylethanolamines; PG, phosphatidylglycerol; PI, phosphatidylinositol; SM, sphingomyelin; VIG-EX, vigorous-intensity exercise group.

To further explore differences between groups, we conducted a PLS-DA analysis, which revealed a clear separation in the changes of the lipid species signature, particularly between the CON and MOD-EX groups (Fig. 1e; performance = 0.63). The top lipid classes contributing most to group separation were glycerophospholipids and TGs, particularly those containing fatty acids with 18 carbon chains (VIP scores > 2.0; Fig. 1f). However, no clear separation in the changes of the lipid species signature was observed between the MOD-EX and VIG-EX groups (Fig. 1e). We also observed significant differences in 124 lipid species plasma levels across 10 subclasses among the three groups, with glycerophospholipids and lysophospholipids being the most prominently affected (Fig. 2). Following the 24-week exercise intervention, the MOD-EX group exhibited a statistically significant increase in the plasma levels of 34 PE species, including alkyl and alkenyl substituent PEs (i.e., PE-O and PE-P). The latter analytes accounted for 30.6% of detected PE species (PE-O: 40.9%; PE-P: 27.8%), compared to the CON group (all P > 0.05; Fig. 2a). In addition, the MOD-EX group displayed a statistically significant increase in PE-O and PE-P levels compared to the VIG-EX group (PE-O: 27.3%; PE-P: 16.7%; all P > 0.05; Fig. 2a).

Similarly, the MOD-EX group demonstrated a statistically significant increase in 22 PC species levels (22.7% of the detected PC species), including four alkyl substituent PCs (PC-O), and 6 PI species (12% of the detected PI species) compared to the CON group (all P > 0.05; Fig. 2b). Additionally, the MOD-EX group also showed significant increases in a total of 4 LPC (14.8% of the detected LPC species) and 15 LPE (53.6% of the detected LPE species) species compared to the CON group (all P > 0.05; Fig. 2c). However, we observed no significant differences in these parameters between MOD-EX and VIG-EX groups and between VIG-EX and CON groups, respectively.

### Moderate-intensity exercise training increases triacylglycerol species plasma levels compared to the control group, particularly in women

**Fig. 3:**
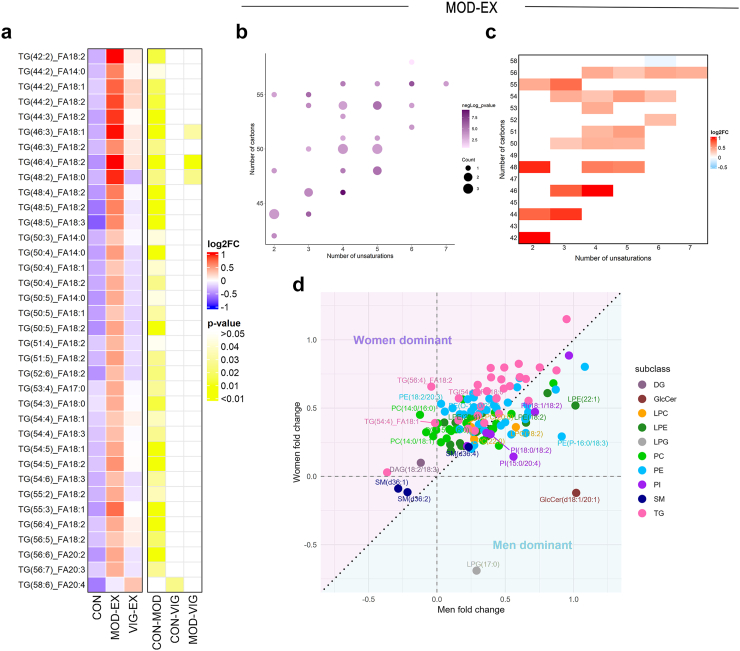
**Effects of 24-week moderate-intensity exercise training on triacylglycerol species and sex-specific lipidomic responses.** a: Heatmap of TG species that significantly changed after the exercise intervention across the three groups. The colour of each square represents the log2 fold change relative to baseline for each lipid and the colour of the yellow square represents the P-value of the comparison between groups. P-values were obtained after one-way ANOVA and Bonferroni correction. b: Bubble plot showing the P-value and the count per TG species categorised by the carbon numbers and unsaturation of the TG carbon chain after 24 weeks of moderate-intensity exercise training. The size of the bubbles represents the number of TGs and the shade of colour represents the negative log10 of P-value, respectively. P-values were obtained from one-way ANOVA and Bonferroni correction. c: Heatmap showing the relative quantification results per TG species categorised by the carbon numbers and unsaturation of the TG after 24-week moderate-intensity exercise training. Data is presented as the log2 fold change (log2FC) relative to baseline. d: Scatter plot showing sex-specific differences in lipidomic responses after intervention in the moderate-intensity exercise group. Each point represents a lipid species coloured by lipid subclass, plotted by its log2 fold change (log2FC) in women (y-axis) and men (x-axis) relative to baseline. Lipids above the diagonal are more enriched in women, while those below are more enriched in men. *Abbreviations*: CON, control group; DAG, diacylglycerol; GlcCer, glucosylceramide; LPC, lysophosphatidylcholine; LPE, lysophosphatidylethanolamine; LPG, lysophosphatidylglycerol; MOD-EX, moderate-intensity exercise group; PC, phosphatidylcholine; PE, phosphatidylethanolamine; PI, phosphatidylinositol; SM, sphingomyelin; TG, triacylglycerol; VIG-EX, vigorous-intensity exercise group.

After 24 weeks of exercise intervention, the MOD-EX group showed a statistically significant increase in the plasma levels of 35 TG species, accounting for 12% of detected TG species, compared to the CON group (all P > 0.05; Fig. 3a). To determine whether these changes followed a specific pattern, we investigated the TG levels in the MOD-EX group, based on carbon chain lengths and their unsaturation level. While all significantly increased TG species had at least one unsaturated bond, no clear pattern emerged across the number of unsaturated bonds and carbon chain lengths (Fig. 3b). However, we observed that the plasma levels of TG species with lower saturation and shorter carbon chains increased to a greater extent in the MOD-EX group (i.e., higher log2FC) compared to the levels of those with higher saturations and longer chains in the CON group (Fig. 3c).

We observed that women displayed higher baseline levels of TG species compared to men (Figure S4b). Therefore, we repeated the above-mentioned analysis separately in men and women. We also observed a clear separation in the changes in the lipid species signature, particularly between the CON and MOD-EX groups (Figures S5a and d). However, we observed that, in men, MOD-EX increased mainly glycerophospholipids and lysophospholipids species (i.e., PE, LPE, PC) levels compared to CON (Fig. 3d, Figure S5b and c), whereas in women, the MOD-EX increased glycerophospholipids species levels and TGs species compared to the CON group (Fig. 3d, Figure S5e and f). No significant differences in these parameters were observed between the MOD-EX and VIG-EX groups, as well as between VIG-EX and CON groups.

### Lipid Ontology enrichment analysis identifies lipid species and pathways in response to 24 weeks of exercise training

**Fig. 4:**
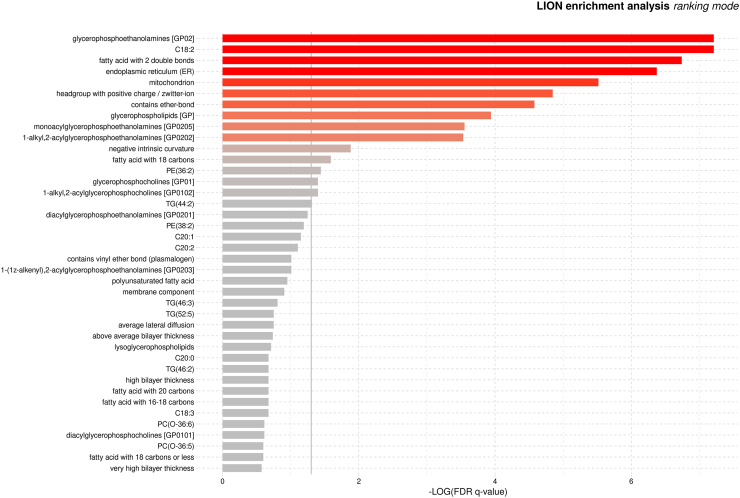
**Identification of lipid species and pathways using Lipid Ontology (LION) enrichment analysis in response to 24 weeks of exercise training based on a three-group comparison.** Statistical analysis included a one-way analysis of variance (ANOVA) F-test for group comparisons and one-tailed Kolmogorov–Smirnov tests to evaluate the distribution of LION terms across the ranked lists. Grey vertical lines indicate the cut-off value for significant enrichments (q < 0.05). Bar colours are scaled according to enrichment levels [−log (FDR q-values)]. *Abbreviations*: FDR, false discovery rate; PC, phosphatidylcholines; PC-O, alkyl substituent phosphatidylcholines; PE, phosphatidylethanolamine; TG, triacylglycerol.

We then used LION-term for the enrichment and pathway analysis to identify the major lipid functional groups and molecular features whose plasma levels changed after 24 weeks of exercise based on a three-group comparison. The analysis revealed that plasma samples were enriched in PEs, lipids containing fatty acids with two double bonds and eighteen carbons (C18:2), lipids containing fatty acids with two double bonds, and lipids related to organelles such as endoplasmic reticulum and mitochondria (Fig. 4). Additionally, the exercise intervention led to significant plasma enrichment in glycerophospholipids, including LPE, PE-O, PC, and PC-O (Fig. 4).

To further explore lipid changes, we performed enrichment analysis on between-group comparisons (i.e., MOD-EX vs. CON, VIG-EX vs. CON, and MOD-EX vs. VIG-EX groups) following the intervention. MOD-EX demonstrated notable enrichment in lipids containing C18:2, fatty acids with 2 double bonds and glycerophosphoethanolamines compared to CON, and in lipids containing ether-bond, TG, and related lipid functions, such as lipid droplets, and lipid storage compared to VIG-EX (Figure S6a and c), respectively. In addition, VIG-EX led to a significant enrichment in the lipids involved in endoplasmic reticulum-related processes, glycerophospholipids, polyunsaturated fatty acids, and fatty acids with 2 double bonds compared to CON group, as well as lipids involved in the plasma membrane, headgroup with negative charge and sphingolipids compared to MOD-EX group (Figure S6b and d), respectively.

### Changes in lipids species after the exercise training intervention are related to the improvements in cardiorespiratory fitness

We previously observed that the 24-week exercise intervention improved cardiorespiratory fitness (VO_2_peak) and reduced adiposity without differences between exercise intensity groups.^20^ Based on these outcomes, we divided the study cohort into responder and non-responder groups based on whether they experienced a clinically meaningful improvement in VO_2_peak (i.e., an increase) and fat mass percentage (i.e., a decrease) after the intervention.

**Fig. 5:**
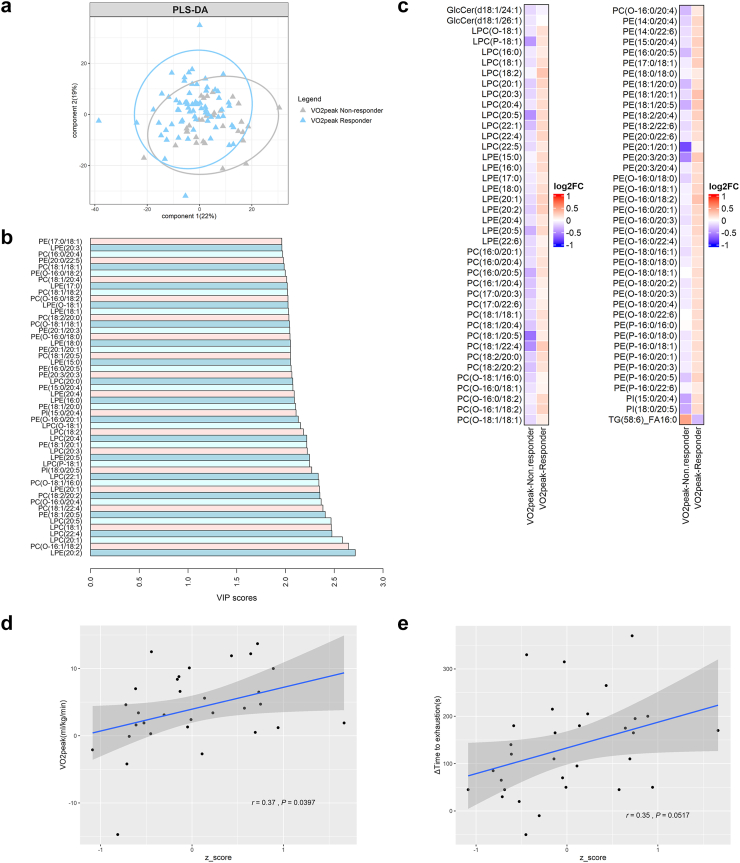
**Changes in lipid species plasma levels following 24 weeks of exercise training are related to the improvements in cardiorespiratory fitness.** a: Partial least square discriminant analysis (PLS-DA) shows the plasma level changes of lipid species between VO_2_ peak responders and non-responders. Data is presented as the log2 fold change (log2FC) relative to the baseline. b: Variable importance in projection (VIP) plot displays the top 50 most important lipids features identified by PLS-DA. c: Heatmap showing the significant difference after the intervention in lipid species levels between VO_2_ peak responders and non-responders. The colour of each square represents the log2 fold change relative to the baseline for each lipid. Analysis of Covariance (ANCOVA) was used to compare the two groups with the group as a covariate. (d and e): Scatter plots showing the correlations between the mean z-score of the changes in lipid species levels in the moderate-intensity exercise group and the change in VO_2_ peak (ml/kg/min) (d) and the change in time to exhaustion (seconds) (e). Pearson correlation coefficients (r) and P-values are provided. The z-score was calculated for each participant based on lipid species that showed significant increases following the intervention in the moderate-intensity exercise group. *Abbreviations*: Cer, ceramide; LacCer, lactosylceramide; LPC, lysophosphatidylcholine; LPE, lysophosphatidylethanolamine; PC, phosphatidylcholine; PC-O, alkyl substituent phosphatidylcholines; PE, phosphatidylethanolamine; PE-O, alkyl substituent phosphatidylethanolamine; PE-P, alkenyl substituent phosphatidylethanolamines; PI, phosphatidylinositol; TG, triacylglycerol.

In terms of cardiorespiratory fitness, we observed a clear separation in lipid species changes signature between responders and non-responders (Fig. 5a, PLS-DA performance = 0.44). The lipid species primarily driving this difference were glycerophospholipids and lysophospholipids (i.e., PE, PC, LPE, and LPC; Fig. 5b). Specifically, cardiorespiratory fitness responders showed significantly greater changes in 35 PE species (including 13 PE-O and 7 PE-P) and 18 PC species (including 6 PC-O) levels compared to non-responders, representing 31.5% and 18.6% of total measured PE and PC, respectively (Fig. 5c). Additionally, responders showed increases in 12 LPC and 9 LPE species levels compared to non-responders (Fig. 5c).

In total, 88 glycerophospholipids showed significant differences in their plasma levels between responders and non-responders, with 11 containing saturated fatty acids (SFAs), 19 containing monounsaturated fatty acids (MUFAs), and 58 containing polyunsaturated fatty acids (PUFAs). These results were consistent when the analysis was repeated within the exercise groups (MOD-EX and VIG-EX) (Figure S7). Additionally, in the MOD-EX group, changes in lipid species levels (mean z-score of all significantly increased species) were positively correlated with improvements in VO_2_peak and time to exhaustion (Fig. 5d and e). Similar results were observed when including sex as a covariate in the correlation analysis (data not shown).

For fat mass percentage, we also identified a separation in the changes of the lipid species signature between responders and non-responders (Figure S8a, PLS-DA performance = 0.51). Key lipids driving this separation included TG, SM, PC, and PE species (Figure S8b). Specifically, fat mass percentage responders showed a decrease in 4 PE and 2 PS species levels, along with an increase in 3 PC and 2 LPC species levels after the intervention (Figure S8c). However, these changes were relatively modest compared to those observed in VO_2_ peak responders.

In addition, we detected a total of 61 participants overlapping between the cardiorespiratory fitness and adiposity responders. In this sense, changes in lipid species associated with the responder status were largely similar across both criteria (Figure S9).

### Post-hoc power and sensitivity analyses

Between-group comparisons across the three intervention groups (CON: n = 35, MOD-EX: n = 32, VIG-EX: n = 34), effect sizes ranged from f = 0.53 to 0.60, with power values exceeding 99% for all selected lipids. In the analysis of sex differences within the MOD-EX group (men: n = 8, women: n = 24), effect sizes ranged from d = 0.27 to 0.84, but statistical power was limited (10–51%) due to the small number of male participants. For the comparison between VO_2_ peak responders (n = 68) and non-responders (n = 31), effect sizes ranged from d = 0.63 to 0.77, yielding power values between 82% and 94% (Table S3).

To assess the robustness of the findings, we conducted a sensitivity analysis by comparing our primary imputation method, QRILC, with alternative approaches, including KNN and half-minimum imputation, and the results remained consistent across these methods (data not shown). Additionally, sensitivity analyses were conducted, including only participants who attended more than 80% of the sessions or all participants regardless of adherence to the training program, and the results did not change (data not shown).

## Discussion

Exercise is a powerful modulator of lipid metabolism. While previous research has predominantly focused on changes in classical lipids blood levels, such as total cholesterol or total TG, comprehensive lipidomic studies exploring the broader impact of exercise training remain limited. In this study, we demonstrated that 24 weeks of MOD-EX training, but not VIG-EX training, led to significant increases in glycerophospholipids, lysophospholipids, and TG species compared to the CON group. Notably, we discovered a sex-specific response to MOD-EX, and that changes in glycerophospholipid lipid levels were associated with improvements in cardiorespiratory fitness. Interestingly, we previously observed that 24 weeks of exercise training did not modify classical lipid markers levels (e.g., total TG or cholesterol) but improved cardiorespiratory fitness and reduced adiposity.^20^ Combined with our current results, this suggests that while traditional lipid markers may remain unchanged, exercise might exert its metabolic benefits through the specific modulation of certain lipid subtypes. In addition, these beneficial effects may be mediated through distinct molecular pathways, potentially depending on exercise intensity. These specific changes could contribute to improvements in metabolic health that are not captured by classical lipid measurements, highlighting the need for more comprehensive lipidomic analyses in future exercise studies.

### Role of moderate-intensity exercise training on lipid species modulation

Here, we unveiled that moderate-intensity exercise modulates lipid species levels beyond traditional markers, particularly glycerophospholipids and TG species. During exercise, fats and carbohydrates are oxidised simultaneously as fuel sources, with their relative contributions determined by exercise duration and intensity. Moderate-intensity exercise relies more on fat oxidation as an energy source compared to vigorous-intensity,^31^^,^^32^ which predominantly engages glycolytic pathways to achieve higher energetic demands.^33^ During moderate-intensity exercise, fatty acid breakdown increases, supported primarily by the mobilisation of fatty acids from adipose tissue into the bloodstream.^34^^,^^35^ This process enhances lipid metabolism promoting TGs as a key energy substrate, which may explain the observed results in the MOD-EX group. In contrast, the increased reliance on carbohydrates during vigorous-intensity exercise might suppress lipid utilisation, potentially explaining the fewer lipid species changes, particularly in TGs, observed after 24 weeks of the VIG-EX group. Thus, a similar metabolic benefit (e.g., cardiorespiratory fitness improvement, adiposity reduction) can be achieved with different exercise intensities using different metabolic pathways. We also observed that the VIG-EX elicited a broader range of individual responses, consistent with increased inter-individual variability in physiological or behavioural adaptations. These findings support the notion that vigorous exercise may not produce uniform effects across individuals and highlight the importance of considering individual response profiles in exercise interventions. Future studies should investigate whether the magnitude of lipidomic adaptations is dependent on exercise dose to clarify whether a minimum threshold of stimulus is required to induce changes in lipid metabolism.

Interestingly, our data revealed a higher increase in TG species in women compared to men after MOD-EX. This aligns with evidence that women rely more on lipid oxidation during moderate-intensity exercise than men, who oxidise a slightly higher percentage of carbohydrates (∼4–5%).^36^^,^^37^ This sex-specific difference is influenced by hormonal factors, particularly oestrogen. Oestrogen enhances lipolysis and mobilisation of adipose tissue by promoting free fatty acid oxidation and mitochondrial biogenesis, while also increasing local catecholamines production.^38^^,^^39^ Furthermore, oestrogen supplementation in men has been shown to increase fat oxidation and reduce carbohydrate oxidation during moderate-intensity exercise,^40^^,^^41^ partially explaining the greater number of TG species increased levels in women compared to men under similar exercise conditions. Future studies should aim to include hormone profiling to better understand the potential contribution of hormonal fluctuations—particularly in women—to lipidomic responses to exercise.

In addition to TG species, the MOD-EX group showed significantly increased levels of glycerophospholipids and lysophospholipids species and an enrichment in their pathways, which may contribute significantly to the distinct lipidomic responses. Concurrent training, i.e., endurance and resistance, has been shown to increase intramuscular PC and PE levels,^42^^,^^43^ which is associated with improved skeletal muscle insulin sensitivity.^44^ Therefore, we hypothesise that the glycerophospholipids species increased plasma levels observed in the MOD-EX group could reflect adaptations in skeletal muscle glycerophospholipids and corresponding improvements in skeletal muscle insulin sensitivity. Notably, the LPIN1 gene in blood, a key regulator of TG and phospholipid biosynthesis, is upregulated after acute submaximal endurance exercise,^45^^,^^46^ which partially supports the observed increased levels of TGs and phospholipid species in MOD-EX. Similarly, six weeks of moderate-intensity continuous endurance training increased PC-O and TG blood concentrations and changed DG, PI, and LPC concentrations, activating their biosynthetic pathways after exercise.^47^ Overall, the physiological role of these glycerophospholipids' increased levels after moderate-intensity exercise is still unknown, but may be involved in membrane fluidity and mitochondrial function, optimising energy production for sustained physical activity,^48^ cell signalling pathways related to inflammation, immune response, and tissue repair,^49^^,^^50^ membrane repair and cellular recovery by inducing Ca^2+^ influx after the mechanical strain of exercise.^51^ Future studies should investigate the specific functions of glycerophospholipids to better understand their role in the role of long-term moderate-intensity exercise in promoting cellular resilience and overall well-being.

Pathway analysis revealed significant plasma enrichment in lipids containing linoleic acid (omega-6 fatty acid; C18:2) and lipids containing fatty acids with two double bonds. This finding aligns with preclinical studies in rats showing that regular exercise increases linoleic acid-containing PC levels.^52^ Linoleic acid-containing phospholipids are involved in maintaining membrane structure and fluidity, energy storage, and acting as lipid mediators in cell signalling, particularly in pathways involved in inflammation, immunity, and cellular responses.^53^ Notably, our MOD-EX group showed a decrease in plasma omega-6 oxylipin levels, oxidised metabolites of omega-6 fatty acids that typically exhibit pro-inflammatory effects.^18^ This suggests that exercise may balance lipid incorporation into structural components (such as phospholipids in membranes) and conversion into signalling molecules, modulating inflammatory responses and cell signalling. Further research is required to elucidate the mechanisms underlying this modulation.

### Role of glycerophospholipids in cardiorespiratory fitness improvements after exercise intervention

Cardiorespiratory fitness is a well-established marker of overall health, associated with a reduced risk of chronic diseases, improved metabolic health, and lower mortality.^54^^,^^55^ We identified that changes in glycerophospholipids may contribute to cardiorespiratory fitness improvements following exercise intervention. Notably, around 88% of the glycerophospholipids showing altered levels contained MUFAs and PUFAs. These results align with previous findings that 16 weeks of moderate-intensity concurrent training significantly increased levels of PUFAs in glycerolipids and glycerophospholipids, along with cardiorespiratory fitness improvements.^56^ Glycerophospholipids are critical components of cell and mitochondrial membranes, essential for maintaining integrity and functionality.^57^ Their role in forming the electron transport chain and ATP production supports enhanced VO_2_max and physical performance.^58^ By optimising membrane fluidity and mitochondrial function, glycerophospholipids may improve energy efficiency during exercise, supporting aerobic capacity and endurance.^59^ In addition, glycerophospholipids play an active role in lipid metabolism by promoting fatty acid oxidation during exercise,^57^ contributing to improved metabolic efficiency and energy production. Furthermore, specific glycerophospholipid species (i.e., LPC and LPE) exhibit anti-inflammatory and antioxidant properties,^57^ which can aid in post-exercise recovery and reduce oxidative stress, which is critical for maintaining physical performance over time. Taken together, these findings underscore the role of glycerophospholipids in driving cardiorespiratory fitness improvements and highlight the importance of lipidomic changes in the cardiovascular and metabolic benefits of exercise training.

### Limitations

Despite these novel insights, the study has limitations to consider. The absence of long-term follow-up limits our ability to assess whether the observed lipids concentrations changes persist or fluctuate with continued exercise. Additionally, while we analysed a thousand lipid species, some subclasses were not fully characterised, particularly TGs, where only partial structural details were resolved. This limitation may affect our understanding of TG metabolism in response to exercise training. Furthermore, the study focused on young sedentary adults, limiting generalisability to other populations, such as older adults, (peri)menopaused women, children, and individuals with pre-existing conditions. Finally, the concurrent exercise intervention prevents isolating the specific effects of aerobic and resistance training.

### Conclusion

Twenty-four weeks of moderate-intensity, but not vigorous-intensity, concurrent exercise training increased glycerophospholipids and TG species plasma levels in comparison to the control group. In addition, we discovered a sex-specific response to moderate-intensity concurrent training, and lipidomic changes appear to contribute to cardiorespiratory fitness improvements, emphasising the role of lipidomic adaptations in the benefits of exercise. While traditional lipid markers may remain unchanged, exercise selectively targets specific lipid subtypes potentially driving metabolic health improvements not detectable through conventional lipid profiling. Future research should further investigate the mechanisms of lipid modulation and the specific role of glycerophospholipids in enhancing cardiorespiratory fitness and overall health.

## Contributors

Conceptualisation, YZ, BMT, JRR, LJF; methodology, YZ, ZZ, BMT, XD, IK, FJOP, AH, TH, JRR, LJF; validation, YZ, BMT, IK, CC, ND, AH, TH; formal analysis, YZ, LJF, AK; data collection, BMT, FJOP, JRR, LJF; data curation, YZ, LJF; writing – original draft, YZ, LJF; writing – review and editing, all authors; supervision, JRR, LJF. All authors commented on the manuscript and approved the final version of the manuscript.

## Data sharing statement

The study protocol is provided with this paper. The raw lipidomics data generated during this study have been deposited in the MetaboLights repository and are publicly available as of the date of publication under the accession number MTBLS12179. The clinical data that support the findings of this study are available from the corresponding author upon reasonable request, as the study consists of a high number of participants and outcomes and requires specific knowledge for data interpretation.

## Code sharing

All custom R scripts used for statistical analysis, and visualisation in this study are openly available on GitHub at: https://github.com/zhangy0816auto/ACTIBATE-lipidomics-open-scource-script. The repository includes code, example input files, and documentation to ensure reproducibility.

## Declaration of interests

The authors declare no competing interests.

## Appendix A Supplementary data

Study Protocol
