# Supplementary material for: Moderate-intensity exercise training uniquely modulates circulating lipid species beyond classical lipid levels in humans
Source: eBioMedicine. 2025 Jul 15;118:105849. doi: 10.1016/j.ebiom.2025.105849 (PMC12283555; doi:10.1016/j.ebiom.2025.105849)
Supplement: Tables S1–S3 and Figures S3–S9 [file mmc2.docx]

**Moderate-intensity exercise training uniquely modulates circulating lipid species beyond classical lipid levels in humans**

Yu Zhang^1^, Zhengzheng Zhang^1,¥^, Borja Martinez-Tellez^2,3,4,¥^, Xinyu Di^1^, Alida Kindt^1^, Isabelle Kohler^5^, Francisco J. Osuna-Prieto^2,6^, Charles Clark^1^, Nicolas Drouin^1^, Amy Harms^1^, Thomas Hankemeier^1^, Jonatan R. Ruiz^2,3,8,†,^*, Lucas Jurado-Fasoli^2,5,9,†,^*

^1^ Metabolomics and Analytics Centre, Leiden Academic Centre for Drug Research (LACDR), Leiden University, the Netherlands

^2^ Department of Physical Education and Sports, Faculty of Sports Science, Sport and Health University Research Institute (iMUDS), University of Granada, Carretera de Alfacar s/n, 18071 Granada, Spain

^3^ CIBER de Fisiopatología de la Obesidad y Nutrición (CIBEROBN), Instituto de Salud Carlos III, Madrid, Spain

^4^ Department of Nursing, Physiotherapy and Medicine, SPORT Research Group (CTS-1024), CIBIS Research Center, University of Almería

^5^ Division of BioAnalytical Chemistry, Department of Chemistry and Pharmaceutical Sciences, Amsterdam Institute of Molecular and Life Sciences (AIMMS), Vrije Universiteit Amsterdam, , Amsterdam, the Netherlands

^6^ Hospital Universitari Joan XXIII de Tarragona, Institut d'Investigació Sanitària Pere Virgili (IISPV), Tarragona, Spain

^7^ CIBER de Diabetes y Enfermedades Metabólicas Asociadas (CIBERDEM)-Instituto de Salud Carlos III (ISCIII), 28029, Madrid, Spain

^8^ Instituto de Investigación Biosanitaria, Ibs.Granada, Granada, Spain

^9^ Department of Physiology, Faculty of Medicine, Sport and Health University Research Institute (iMUDS), University of Granada, Granada, Andalucía, Spain

^¥^These authors contributed equally.

^†^These authors shared senior authorship.

*** Corresponding authors**: [ruizj@ugr.es](mailto:ruizj@ugr.es) and [juradofasoli@ugr.es](mailto:juradofasoli@ugr.es)

**SUPPLEMENTARY MATERIAL**

**Table S1**: Number of lipid species detected for each lipid class using the validated hydrophilic interaction chromatography-tandem mass spectrometry (HILIC-MS/MS) platform.

| **Category** | **Lipid classes** | **Number of lipids detected^1^** | **Number of lipids quantified^2^** | **Acquisition method^3^** |
| --- | --- | --- | --- | --- |
| Glycerolipids | DG | 51 | 11 | 2 |
|  | TG | 446 | 292 | 3 |
| Glycerophospholipids | PC | 109 | 97 | 1 |
|  | PE | 153 | 111 | 3 |
|  | PS | 55 | 26 | 1 |
|  | PI | 65 | 50 | 1 |
|  | PG | 66 | 12 | 1 |
|  | LPC | 31 | 27 | 1 |
|  | LPE | 43 | 28 | 1 |
|  | LPS | 18 | 2 | 1 |
|  | LPI | 19 | 6 | 1 |
|  | LPG | 20 | 7 | 1 |
| Sphingolipids | Cer | 12 | 12 | 2 |
|  | SM | 71 | 68 | 2 |
|  | HexCer | 23 | 17 | 2 |
|  | LacCer | 11 | 10 | 2 |
|  | GlcCer | 8 | 8 | 2 |
| Sterols | CE | 42 | 10 | 2 |

*Abbreviations:* CE, cholesterol ester; Cer, ceramide; DG, diacylglycerol; GlcCer, glucosylceramide; HexCer, hexosylceramide; LacCer, lactosylceramide; LPC, lysophosphatidylcholine; LPE, lysophosphatidylethanolamine; LPG, lysophosphatidylglycerol; LPI, Lysophosphatidylinositol; LPS, Lysophosphatidylserine; PC, phosphatidylcholines; PE, phosphatidylethanolamine; PG, phosphatidylglycerol; PI, phosphatidylinositol; PS, phosphatidylserine; SM, sphingomyelin; TG, triacylglycerol.

**^1^**Number of lipids detected: number of lipid species in each class detected and identified with the HILIC-MS/MS platform.

**^2^**Number of lipids quantified: number of lipid species in each class used for data analysis after applying quality control check.

**^3^**Acquisition method: electrospray ionization (ESI) mode used for data acquisition, i.e., 1, ESI negative mode; 2, ESI positive mode; and 3, ESI positive mode for TG and negative mode for PE, respectively.

**Table S2**: Effect of the 24-week supervised exercise intervention on classical lipid markers plasma levels across the three training groups.

|  | **CON (n=34)** | | | | | | **MOD-EX (n=32)** | | | | | | **VIG-EX (n=34)** | | | | | |  |
| --- | --- | --- | --- | --- | --- | --- | --- | --- | --- | --- | --- | --- | --- | --- | --- | --- | --- | --- | --- |
|  | **Baseline** | | **Post** | | **∆ (Post-baseline)** | | **Baseline** | | **Post** | | **∆ (Post-baseline)** | | **Baseline** | | **Post** | | **∆ (Post-baseline)** | | ***P*-value** |
|  | Mean | SD | Mean | SD | Mean | SD | Mean | SD | Mean | SD | Mean | SD | Mean | SD | Mean | SD | Mean | SD |  |
| TC (mg/dL) | 154.1 | 30.0 | 162.8 | 36.9 | 8.7 | 22.7 | 164.4 | 30.5 | 166.3 | 27.8 | 1.9 | 21.6 | 170.9 | 31.7 | 173.1 | 34.5 | 2.2 | 22.2 | 0.801 |
| HDL-C (mg/dL) | 53.5 | 9.6 | 55.0 | 10.4 | 1.5 | 9.1 | 52.2 | 11.9 | 56.3 | 10.5 | 4.1 | 9.1 | 51.9 | 13.3 | 56.3 | 13.2 | 4.4 | 8.1 | 0.351 |
| LDL-C (mg/dL) | 85.9 | 25.5 | 92.9 | 29.0 | 7.0 | 14.8 | 96.7 | 28.0 | 92.4 | 21.6 | -4.3 | 20.7 | 100.9 | 23.7 | 98.2 | 25.6 | -2.6 | 18.5 | 0.222 |
| TG (mg/dL) | 74.1 | 34.7 | 75.6 | 39.8 | 1.6 | 27.2 | 88.4 | 63.0 | 88.6 | 55.1 | 0.2 | 28.7 | 90.9 | 47.4 | 94.8 | 50.3 | 3.8 | 35.1 | 0.712 |
|  | **CON (n=14)** | | | | | | **MOD-EX (n=11)** | | | | | | **VIG-EX (n=13)** | | | | | |  |
| APOA1 (mg/dL) | 150.5 | 23.9 | 155.1 | 21.5 | 4.6 | 16.2 | 140.4 | 14.3 | 152.5 | 18.8 | 12.1 | 14.8 | 135.3 | 23.4 | 147.8 | 20.5 | 12.5 | 17.9 | 0.813 |
| APOB (mg/dL) | 68.6 | 17.2 | 61.8 | 15.0 | -6.8 | 16.4 | 72.8 | 20.1 | 66.0 | 17.3 | -6.8 | 18.0 | 75.6 | 29.3 | 69.2 | 26.1 | -6.4 | 11.3 | 0.875 |

*Abbreviations:* APOA1, apolipoprotein A-I; APOB, apolipoprotein B; CON, control group; HDL-C, high-density lipoprotein cholesterol; LDL-C, low-density lipoprotein cholesterol; MOD-EX, moderate-intensity exercise group; TC, total cholesterol; TG, total triglycerides; VIG-EX, vigorous-intensity exercise group. The *p*-values were obtained with analyses of covariance (ANCOVA) adjusting for baseline values.

**Table S3:** Post hoc power analysis for selected lipid species across key comparisons.

| Lipid | Analysis | Sample size | Effect Size | Power |
| --- | --- | --- | --- | --- |
| TG(46:4)_FA18:2 | One-way ANOVA (3 groups) | CON:35  MOD-EX: 32  VIG-EX:34 | 0.602092756 | 0.999863 |
| PC(18:2/18:2) |  |  | 0.58818921 | 0.999769 |
| PI(18:1/18:2) |  |  | 0.555954661 | 0.999284 |
| PE(18:1/18:2) |  |  | 0.532518988 | 0.998459 |
| LPE(20:2) |  |  | 0.531014583 | 0.998384 |
| GlcCer(d18:1/20:1) | Sex difference (MOD group)  Two-sample t-test | Women:24  Men:8 | 0.701965079 | 0.383943 |
| TG(56:4)_FA18:2 |  |  | 0.531490414 | 0.242742 |
| PE(18:2/20:3) |  |  | 0.270659914 | 0.098389 |
| PC(14:0/16:0) |  |  | 0.510019601 | 0.227254 |
| LPG(17:0) |  |  | 0.83975117 | 0.512387 |
| LPE(20:2) | Responder vs. Non-responder  Two-sample t-test | Responder: 68  Non-responder:31 | 0.76906046 | 0.939843 |
| LPC(20:1) |  |  | 0.727347332 | 0.913545 |
| PE(18:1/20:5) |  |  | 0.67257246 | 0.867082 |
| PC(18:1/22:4) |  |  | 0.665518135 | 0.860036 |
| PI(18:0/20:5) |  |  | 0.630472948 | 0.821316 |

*Abbreviations:* ANOVA, analysis of variance; CON, control group; GlcCer, glucosylceramide; LPC, lysophosphatidylcholine; LPE, lysophosphatidylethanolamine; LPG, lysophosphatidylglycerol; MOD-EX, moderate-intensity exercise group; PC, phosphatidylcholine; PE, phosphatidylethanolamine; PI, phosphatidylinositol; TG, triacylglycerol; VIG-EX, vigorous-intensity exercise group.


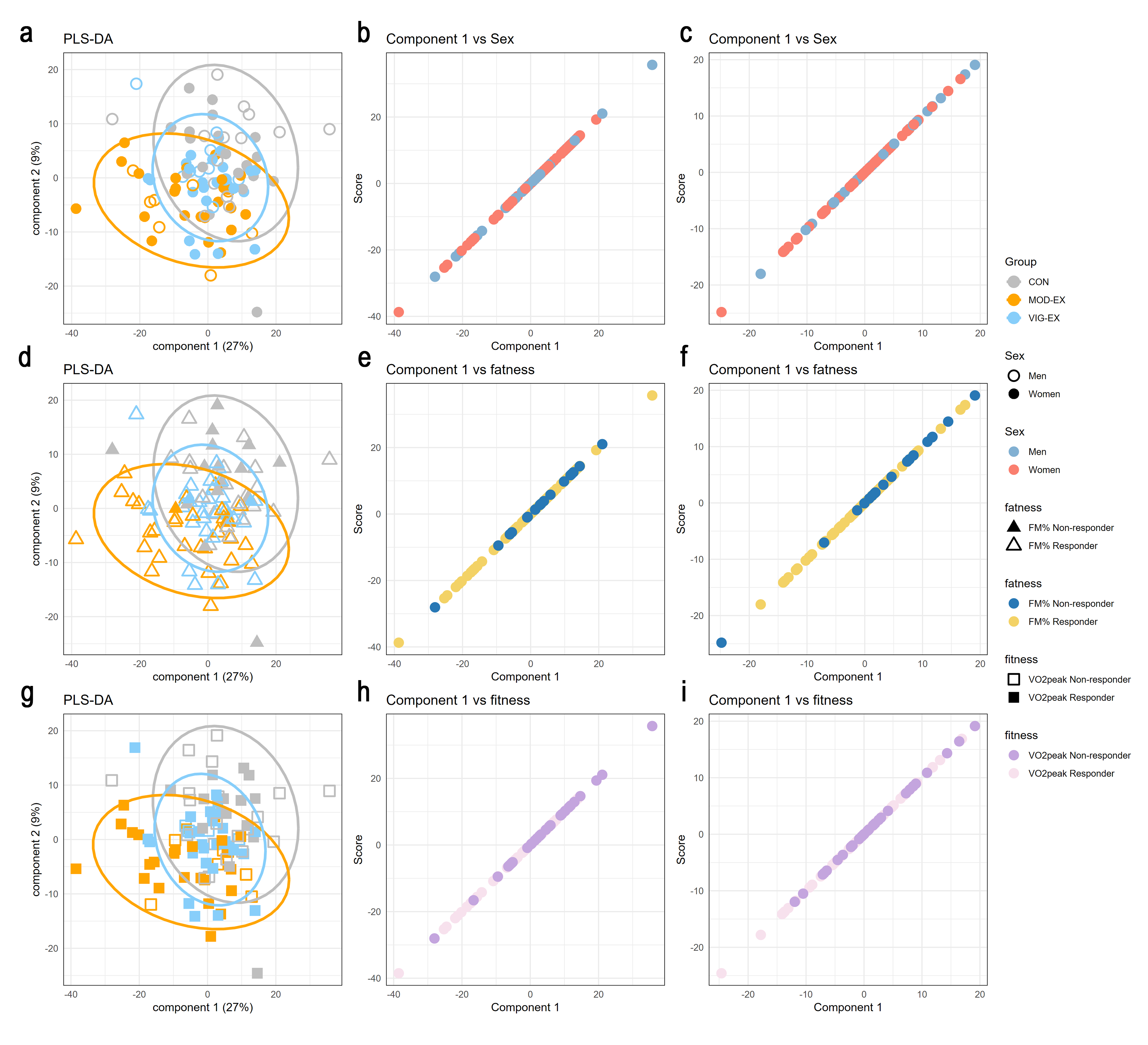


**Figure S3.** Partial least squares discriminant analysis (PLS-DA) of plasma lipidomic profiles by sex, fat mass response, and fitness response.

(a, d, g) Partial least squares discriminant analysis (PLS-DA) plots showing separation of the three intervention groups based on plasma lipid species. Different symbols indicate participant characteristics:

• (a) Sex: hollow circles (men), solid circles (women);

• (d) Fat mass percentage responder status: hollow triangles (responders), solid triangles (non-responders);

• (g) VO₂peak relative to body weight responder status: solid squares (responders), hollow squares (non-responders).

(b, c, e, f, h, i) Scatter plots displaying Component 1 versus Component 2 scores to evaluate the contributions of sex (b, c), fat mass percentage (e, f), and VO₂peak fitness (h, i) to the observed variance.

Abbreviations: CON, control group; MOD-EX, moderate-intensity exercise group; VIG-EX, vigorous-intensity exercise group.





**Figure S4.** Baseline and intervention-related differences in plasma lipidomic profiles.

(a) Principal component analysis (PCA) illustrating the lipidomic distribution between participants in the vigorous-intensity exercise group before (blue triangles) and after (red circles) the intervention.

(b) Volcano plot showing baseline sex differences in plasma lipid species. Lipid species are colored by lipid subclass, and the dashed line represents the significance threshold (p = 0.05).

Abbreviations: GlcCer, glucosylceramide; LPE, lysophosphatidylethanolamine; PE, phosphatidylethanolamine; PI, phosphatidylinositol; TG, triacylglycerol.





**Figure S5.** Effects of 24 weeks of exercise training on lipid species plasma levels in men and women.

(a, d) Partial least square discriminant analysis (PLS-DA) illustrating sample classification in men (a) and women (d). Data is presented as the log2 fold change (log2FC) relative to the baseline.

(b, e) Variable importance in projection (VIP) plot highlighting the top 50 most important lipids features identified by PLS-DA in men (b) and women (e).

(c, f) Heatmap of lipid species whose plasma levels significantly changed after exercise training in men (c) and women (f). The color of each square represents the log2 fold change relative to the baseline for each lipid (left column) and the color of the yellow square represents the p-value of the comparison between the groups (right column). P-values were obtained after one-way ANOVA and Bonferroni correction.

*Abbreviations*: CON, control group; DAG, diacylglycerol; GlcCer, glucosylceramide; LPC, lysophosphatidylcholine; LPE, lysophosphatidylethanolamine; LPG, lysophosphatidylglycerol; MOD-EX, moderate-intensity exercise group; PC, phosphatidylcholine; PC-O, alkyl substituent phosphatidylcholines; PE, phosphatidylethanolamine; PE-O, alkyl substituent phosphatidylethanolamine; PE-P, alkenyl substituent phosphatidylethanolamines; PI, phosphatidylinositol; SM, sphingomyelin; VIG-EX, vigorous-intensity exercise group; TG, triacylglycerol.


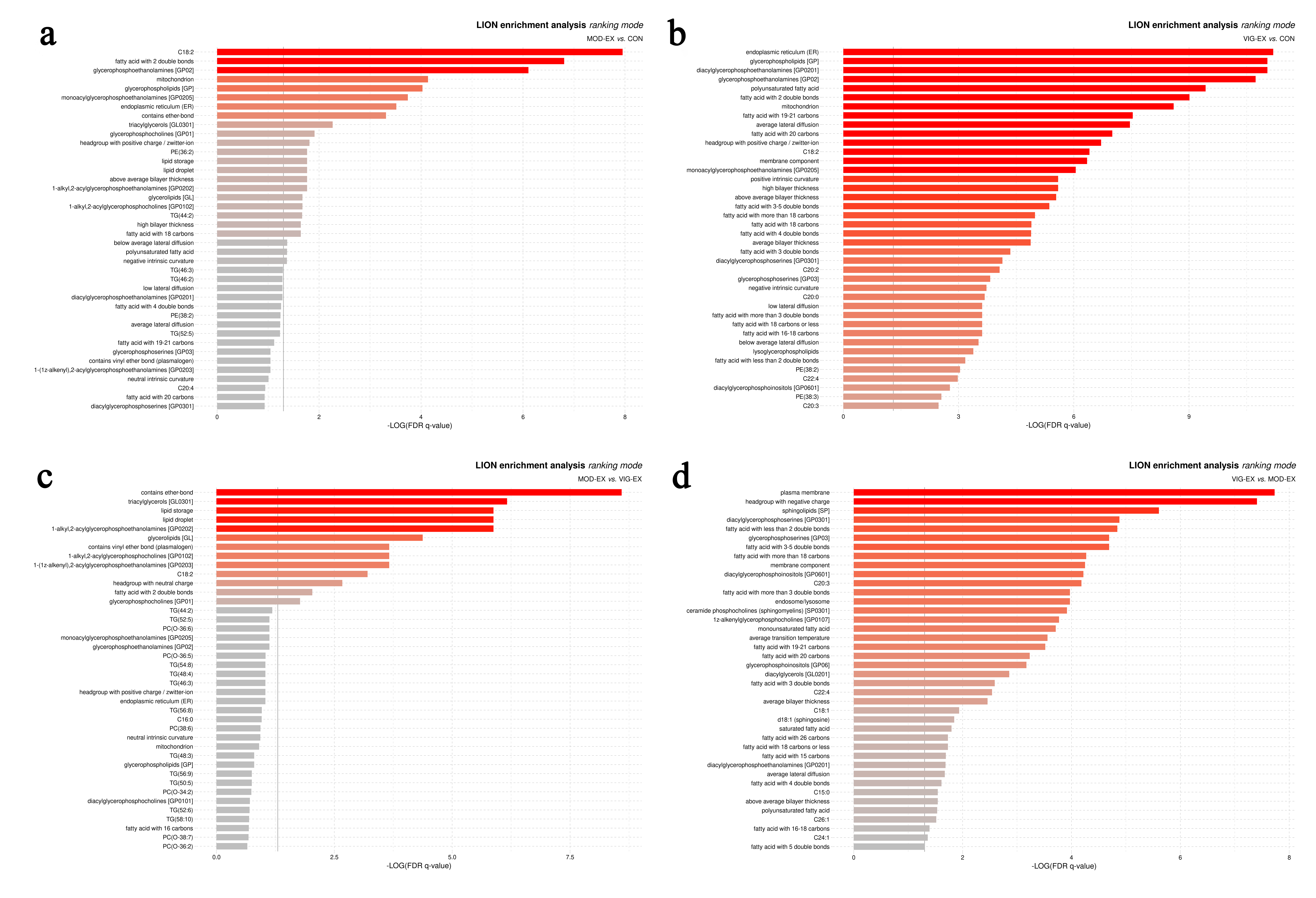
**Figure S6.** Lipid Ontology (LION) enrichment analysis highlighting lipid species and pathways responsive to 24 weeks of exercise training, with comparisons between MOD-EX vs. CON (a), VIG-EX vs. CON (b), MOD-EX vs. VIG-EX (c) and VIG-EX vs. MOD-EX (d) groups, respectively.

Statistical analysis included one-tailed Welch's t-tests for group comparisons and one-tailed Kolmogorov-Smirnov tests to evaluate the distribution of LION terms across the ranked lists. Enrichment indicates terms associated with the first group. Gray vertical lines indicate the cut-off value for significant enrichments (*q* < 0.05). Bar colors are scaled according to enrichment levels [−log (FDR q-values)].

*Abbreviations:* CON, control group; FDR, false discovery rate; MOD-EX, moderate-intensity exercise group; PC, phosphatidylcholines; PC-O, alkyl substituent phosphatidylcholines; PE, phosphatidylethanolamine; TG, triacylglycerol; VIG-EX, vigorous-intensity exercise group.


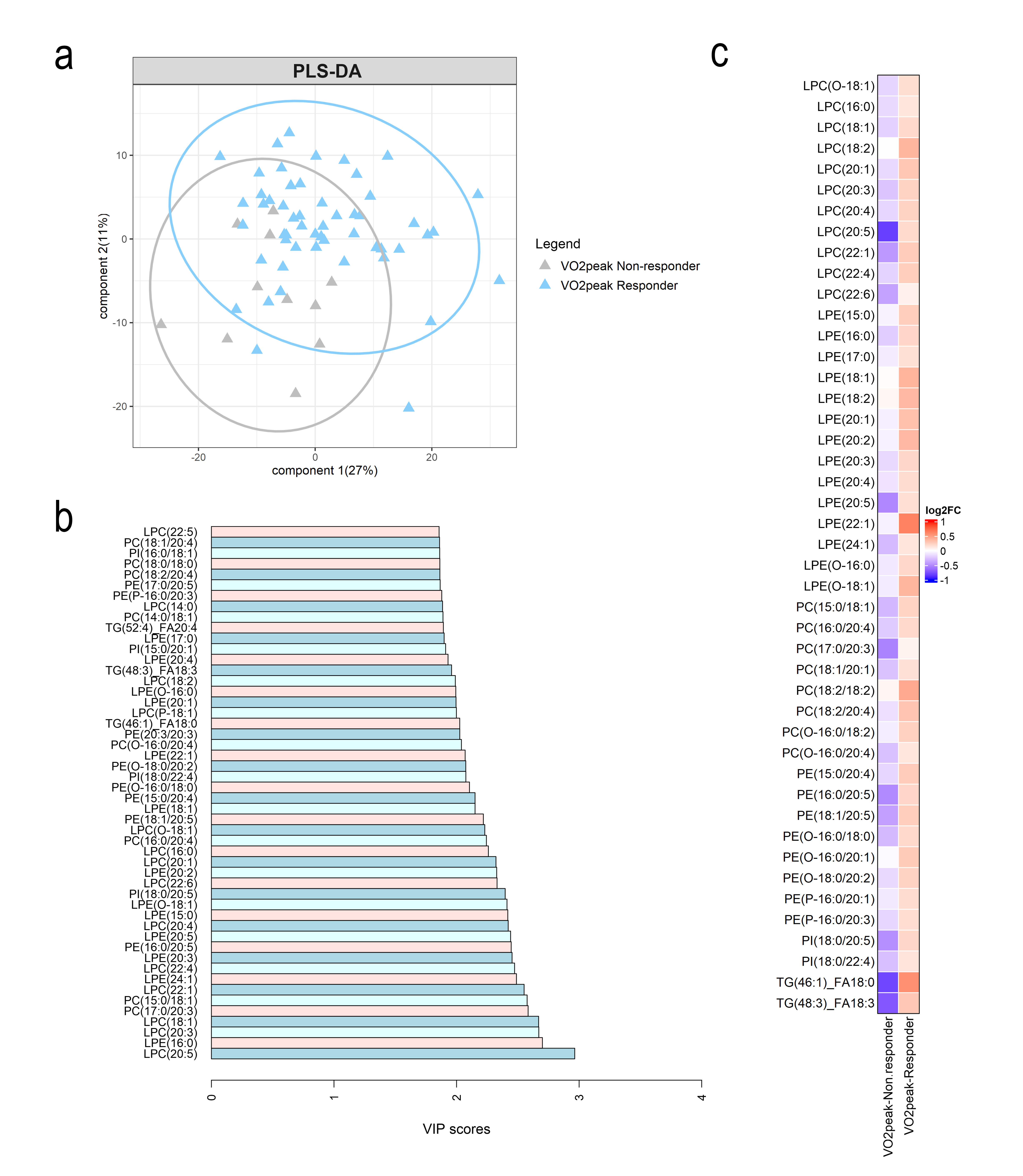


**Figure S7.** Differences in plasma levels changes in lipid species between cardiorespiratory fitness responders and non-responders in the MOD-EX and VIG-EX groups.

(a): Partial least square discriminant analysis (PLS-DA) representing the separation between responders and non-responders groups for VO_2_peak in the MOD-EX and VIG-EX groups. Data is presented as the log2 fold change (log2FC) relative to the baseline. Sample sizes: VO_2_peak Non-responders: 11; VO_2_peak responders: 53.

(b): Variable importance in projection (VIP) plot displaying the top 50 most important lipids features identified by PLS-DA in VO_2_peak.

(c): Heatmap showing lipids that significantly differ between responders and non-responders for VO_2_peak. The color of each square represents the log2 fold change relative to the baseline for each lipid. Analysis of covariance (ANCOVA) was used to compare the two groups with the group as a covariate.

*Abbreviations*: LPC, lysophosphatidylcholine; LPC-O, alkyl substituent lysophosphatidylcholine; LPE, lysophosphatidylethanolamine; LPE-O, alkyl substituent lysophosphatidylethanolamine; LPG, lysophosphatidylglycerol; PC, phosphatidylcholine; PC-O, alkyl substituent phosphatidylcholines; PE, phosphatidylethanolamine; PE-O, alkyl substituent phosphatidylethanolamine; PE-P, alkenyl substituent phosphatidylethanolamines; PI, phosphatidylinositol; PS, phosphatidylserine; TG, triacylglycerol.





**Figure S8.** Differences in plasma levels of lipid species between fat mass responders and non-responders.

a: Partial least square discriminant analysis (PLS-DA) represents separation in fat mass responder and Non-responder. Data is presented as the log2 fold change (log2FC) relative to the baseline.

b: Variable importance in projection (VIP) plot displaying the top 50 most important lipids features identified by PLS-DA.

c: Heatmap showing lipids that significantly differ between fat mass responders and non-responders. The color of each square represents the log2 fold change relative to the baseline for each lipid. Analysis of covariance (ANCOVA) was used to compare the two groups with the group as a covariate.

*Abbreviations*: Cer, ceramide; LPC, lysophosphatidylcholine; LPE, lysophosphatidylethanolamine; LPG, lysophosphatidylglycerol; PC, phosphatidylcholine; PE, phosphatidylethanolamine; PI, phosphatidylinositol; PS, phosphatidylserine; SM, sphingomyelin; TG, triacylglycerol.





**Figure S9**. Differences in plasma levels of lipid species between overlapping fitness and fatness responders and non-responders.

(a): Partial least square discriminant analysis (PLS-DA) representing the separation between overlapping responders and non-responders groups for VO2peak. Data is presented as the log2 fold change (log2FC) relative to the baseline. Sample sizes: Non-responders: 38; responders: 61.

(b): Variable importance in projection (VIP) plot displaying the top 50 most important lipids features identified by PLS-DA in overlap.

(c): Heatmap showing lipids that significantly differ between overlapping responders and non-responders. The color of each square represents the log2 fold change relative to the baseline for each lipid. Analysis of covariance (ANCOVA) was used to compare the two groups with the group as a covariate.

Abbreviations: DAG, diacylglycerol; GlcCer, glucosylceramide; LPC, lysophosphatidylcholine; LPE, lysophosphatidylethanolamine; PC, phosphatidylcholine; PC-O, alkyl substituent phosphatidylcholines; PE, phosphatidylethanolamine; PE-O, alkyl substituent phosphatidylethanolamine; PE-P, alkenyl substituent phosphatidylethanolamines; PI, phosphatidylinositol; SM, sphingomyelin; TG, triacylglycerol.
