# Supplementary material for: Moderate-intensity exercise training uniquely modulates circulating lipid species beyond classical lipid levels in humans
Source: eBioMedicine. 2025 Jul 15;118:105849. doi: 10.1016/j.ebiom.2025.105849 (PMC12283555; doi:10.1016/j.ebiom.2025.105849)
Supplement: Figures S1 and S2 [file mmc3.docx]

**Moderate-intensity exercise training uniquely modulates circulating lipid species beyond classical lipid levels in humans**

Yu Zhang^1^, Zhengzheng Zhang^1,¥^, Borja Martinez-Tellez^2,3,4,¥^, Xinyu Di^1^, Alida Kindt^1^, Isabelle Kohler^5^, Francisco J. Osuna-Prieto^2,6^, Charles Clark^1^, Nicolas Drouin^1^, Amy Harms^1^, Thomas Hankemeier^1^, Jonatan R. Ruiz^2,3,8,†,^*, Lucas Jurado-Fasoli^2,5,9,†,^*

^1^ Metabolomics and Analytics Centre, Leiden Academic Centre for Drug Research (LACDR), Leiden University, the Netherlands

^2^ Department of Physical Education and Sports, Faculty of Sports Science, Sport and Health University Research Institute (iMUDS), University of Granada, Carretera de Alfacar s/n, 18071 Granada, Spain

^3^ CIBER de Fisiopatología de la Obesidad y Nutrición (CIBEROBN), Instituto de Salud Carlos III, Madrid, Spain

^4^ Department of Nursing, Physiotherapy and Medicine, SPORT Research Group (CTS-1024), CIBIS Research Center, University of Almería

^5^ Division of BioAnalytical Chemistry, Department of Chemistry and Pharmaceutical Sciences, Amsterdam Institute of Molecular and Life Sciences (AIMMS), Vrije Universiteit Amsterdam, , Amsterdam, the Netherlands

^6^ Hospital Universitari Joan XXIII de Tarragona, Institut d'Investigació Sanitària Pere Virgili (IISPV), Tarragona, Spain

^7^ CIBER de Diabetes y Enfermedades Metabólicas Asociadas (CIBERDEM)-Instituto de Salud Carlos III (ISCIII), 28029, Madrid, Spain

^8^ Instituto de Investigación Biosanitaria, Ibs.Granada, Granada, Spain

^9^ Department of Physiology, Faculty of Medicine, Sport and Health University Research Institute (iMUDS), University of Granada, Granada, Andalucía, Spain

^¥^These authors contributed equally.

^†^These authors shared senior authorship.

*** Corresponding authors**: [ruizj@ugr.es](mailto:ruizj@ugr.es) and [juradofasoli@ugr.es](mailto:juradofasoli@ugr.es)


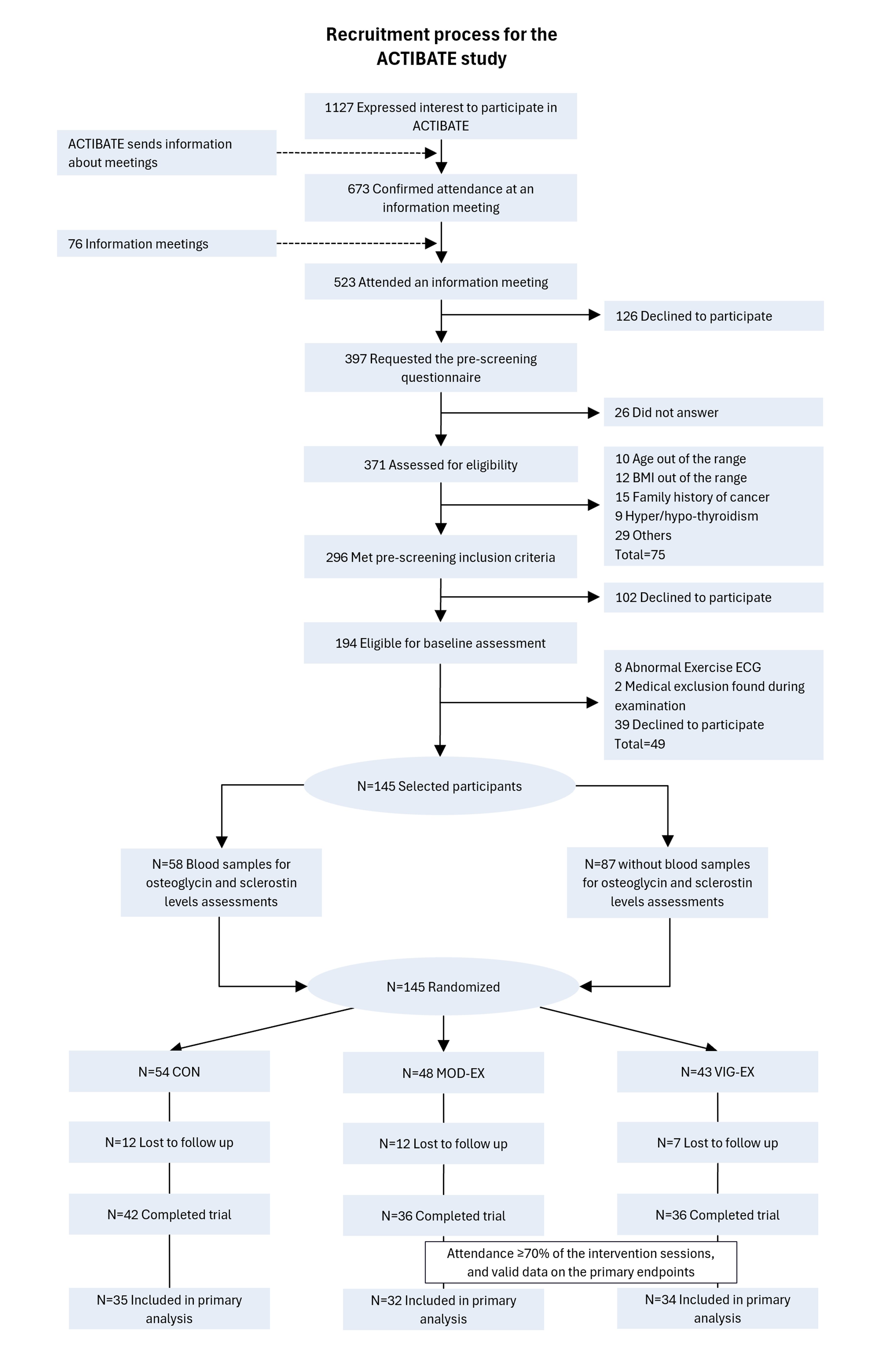


**Figure S1.** Enrollment of study participants in the ACTIBATE study.

*Abbreviations:* BMI, body mass index; CON, control group; ECG, electrocardiogram; MOD-EX, moderate-intensity exercise group; VIG-EX, vigorous-intensity exercise group.





**Figure S2.** Assessment of lipidomics data quality using principal component analysis and lipid class abundance.

(a, b, c) Principal component analysis (PCA) is plotted using quality controls using the three different acquisition methods, i.e., 1 (a), 2 (b), and 3 (c). The quality control samples (black circles) show distinct clustering compared to biosamples, which were measured in three batches (green, blue, and yellow circles), highlighting the good quality of the lipidomics data.

(d) Representative extracted ion chromatogram of standards analytes spiked in plasma, analyzed using the validated HILIC-MS/MS lipidomics platform in both positive (+) and negative (-) ESI acquisition modes.

(e) Relative abundance of the number of lipid classes after having passed the quality control check.

*Abbreviations:* CE, cholesterol ester; Cer, ceramide; DAG, diacylglycerol; GlcCer, glucosylceramide; HexCer, Hexosylceramide; LacCer, Lactosylceramide; LPC, lysophosphatidylcholine; LPE, lysophosphatidylethanolamine; LPG, lysophosphatidylglycerol; LPI, Lysophosphatidylinositol; LPS, Lysophosphatidylserine; PC, phosphatidylcholine; PE, phosphatidylethanolamine; PG, phosphatidylglycerol; PI, phosphatidylinositol; PS, Phosphatidylserine; SM, sphingomyelin; SQC, statistical quality control; TG, triacylglycerol.
